# Proposal of a vertically polarized superconducting multipole wiggler using Nb_3_Sn coils

**DOI:** 10.1107/S1600577525004382

**Published:** 2025-06-17

**Authors:** Hirotoshi Saito, Kimichika Tsuchiya, Chikaori Mitsuda, Kentaro Harada, Yoshito Shimosaki, Takashi Obina

**Affiliations:** a High Energy Accelerator Research Organization (KEK) 1-1 Oho Tsukuba Ibaraki305-0801 Japan; Tohoku University, Japan

**Keywords:** vertically polarized hard X-ray source, V-SC-MPW, superconducting multipole wiggler using Nb_3_Sn coils

## Abstract

We propose a vertically polarized superconducting multipole wiggler (V-SC-MPW) that enables the use of vertically polarized hard X-rays with minimal impact on beam quality. Vertical polarization facilitates unique experimental setups by allowing horizontal arrangement of optical equipment, which is difficult to realize with horizontally polarized X-rays. However, significant emittance growth has prevented the adoption of such vertically polarized, high-field devices in third-generation light sources. To address this challenge, the V-SC-MPW employs a short-period design. By utilizing Nb_3_Sn superconducting wires, which have a critical current density approximately 10 times higher than that of conventional NbTi, the period length can be shortened while maintaining the required magnetic field, thereby reducing the beam orbit amplitude and the resulting emittance growth. A case study that considers the introduction of the V-SC-MPW into PF-HLS, a future light source planned at KEK, shows that, with a horizontal magnetic gap of 30 mm, a design featuring a peak magnetic field of 2.44 T, a period length of 85 mm and an orbit amplitude of 54 µm at a beam energy of 2.5 GeV is feasible. This seven-period V-SC-MPW, assumed to be installed in a non-achromatic straight section of PF-HLS, is estimated to result in emittance growths of 15.6 pm rad horizontally and 1.0 pm rad vertically. These minimal impacts indicate that the short-period V-SC-MPW is a promising insertion device for utilizing vertically polarized hard X-rays in modern low-emittance rings with intermediate beam energies.

## Abstract 2

We propose a vertically polarized superconducting multipole wiggler (V-SC-MPW) designed to reduce emittance growth. This paper presents a low-field, short-period design utilizing Nb_3_Sn coils, demonstrating its feasibility for vertically polarized hard X-ray generation in modern low-emittance rings with minimal impact on beam quality.

## 1. Introduction

In many synchrotron radiation facilities with an intermediate beam energy (typically 2–3 GeV), wigglers with peak magnetic fields approximately 2 T or higher along the central beam orbit are used to produce high-energy X-rays. Third-generation light sources use superconducting or permanent magnet wigglers for practical applications. As superconducting insertion devices (IDs), high-field wavelength shifters (SC-WLSs) (Borovikov *et al.*, 2001 ▸; Chang *et al.*, 2005 ▸; Klysubun *et al.*, 2007 ▸) and multipole wigglers (SC-MPWs) (Berger *et al.*, 2001 ▸; Mezentsev *et al.*, 2018 ▸) with peak magnetic fields exceeding 6 T have been introduced mainly in low-energy rings below 2 GeV. SC-MPWs with peak magnetic fields about 3–4 T have also been widely used mainly in 3 GeV-class rings (Khrushchev *et al.*, 2006 ▸; Tanabe *et al.*, 2022 ▸). As permanent magnet IDs, multipole wigglers with peak magnetic fields of approximately 2 T have been used. In addition to conventional out-vacuum multipole wigglers (Kim *et al.*, 2001 ▸; Kulesza *et al.*, 2008 ▸), in-vacuum ones have also emerged in recent years (Marcouille *et al.*, 2013 ▸; Marcouille *et al.*, 2019 ▸).

In such wigglers for high-energy X-rays, a vertical gap and horizontal polarization are generally adopted. This is because creating a narrow aperture in the horizontal direction is difficult, making it less effective for generating high magnetic fields in the case of horizontal off-axis beam injection, which is commonly employed in storage rings. In contrast, a superconducting wavelength shifter with a horizontal gap and vertical polarization (V-SC-WLS) has been used for over 40 years since 1983 at the Photon Factory (PF), a synchrotron radiation facility at KEK (Yamakawa *et al.*, 1986 ▸; Ohmi *et al.*, 1992 ▸). Vertical polarization allows optical equipment to be arranged horizontally, enabling more diverse experimental systems. At the PF beamline, a highly stable, large imaging system for a separated two-crystal X-ray interferometer has been realized by implementing sophisticated vibration suppression mechanisms, which are difficult to incorporate with vertical optical systems (Yoneyama *et al.*, 2013 ▸). Due to the limited size and operating spectral range of phase shifters in the hard X-ray region, such vertically polarized wigglers are considered highly beneficial.

Against this advantage, we also need to consider that superconducting wigglers with vertical polarization tend to have a significant impact on beam emittance due to quantum excitation by photon emissions. This arises from the difficulty in generating high magnetic fields with a horizontal gap, resulting in a longer magnet length (period length) and a larger orbit amplitude of the beam. For this reason, vertically polarized superconducting wigglers have not been adopted in low-emittance third-generation light sources. Such an impact on emittance is also a significant issue in the next-generation light source plan at KEK, PF-HLS (Photon Factory Hybrid Light Source) (High Energy Accelerator Research Organization, 2024 ▸). If the existing V-SC-WLS with a peak magnetic field of 4.8 T, a pole length of 200 mm and an orbit amplitude of 6 mm from the beam axis is introduced into PF-HLS with a beam energy of 2.5 GeV, a horizontal emittance of 208 pm rad and a circumference of 750 m, the estimated vertical emittance growth reaches several hundred picometre radians. To enable the use of vertically polarized hard X-rays in PF-HLS and other low-emittance rings in the future, it is important to significantly reduce this impact on emittance.

In this paper, we propose a vertically polarized superconducting multipole wiggler (V-SC-MPW) using Nb_3_Sn as a source for vertically polarized hard X-rays with minimal impact on beam emittance. This ID aims to reduce the orbit amplitude of the beam, which leads to emittance growth, by minimizing the period length and magnetic field. The multipole type, which can increase photon flux through the number of poles, helps to reduce the required magnetic field, and the high critical current density of Nb_3_Sn contributes to shortening the period length necessary to generate the required field. In this study, assuming its introduction to PF-HLS, we estimated the achievable period length and magnetic field in this scheme and the impact on emittance. The conceptual design of this device is discussed in Section 2, followed by analyses of the magnetic field characteristics in Section 3 and an evaluation of the impact on beam emittance in Section 4.

## 2. Conceptual design of V-SC-MPW

**Figure 1:**
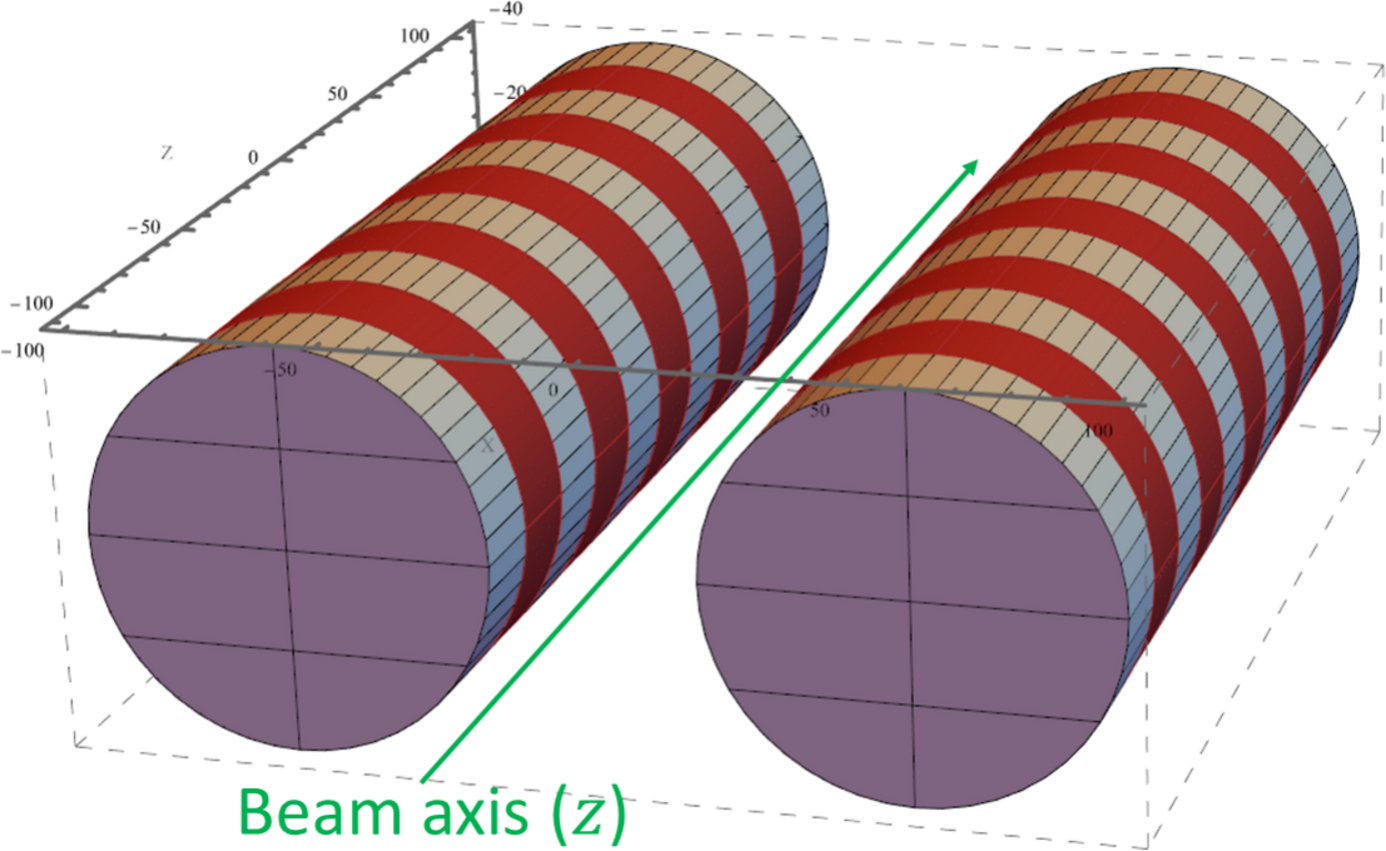
3D model of a V-SC-MPW with the vertical circular winding coils generated by a *RADIA* calculation.

The required features and target parameters of the V-SC-MPW discussed in this study are summarized in Table 1 ▸. These design parameters are selected based on the specifications of PF-HLS, whose main parameters relevant to this study are shown in Table 2 ▸. The peak magnetic field on the central beam orbit was set to 2–3 T, which is the minimum required for generating hard X-rays. By reducing the period length within the range in which this magnetic field can be achieved, the orbit amplitude is minimized. The target values of the period length and the orbit amplitude were determined based on the calculation results presented later. The number of periods was limited to fewer than 10, which corresponds to the magnet length of less than 1 m, to enable installation in the 2 m straight section of PF-HLS. This compact design also contributes to reducing emittance growth and thermal load on the beamline. Given the relatively large 30 mm gap assumed in PF-HLS, which is necessary for the off-axis injection scheme adopted to support high-current operation, it is difficult to generate a magnetic field of 2–3 T using permanent magnets. Therefore, superconducting magnets must be used. Conventionally, NbTi coils have been used for superconducting-based IDs. Among superconducting materials with higher critical current densities, Nb_3_Sn has approximately ten times the critical current density of NbTi and is progressing toward practical application. Its significantly higher critical current density is beneficial for achieving a shorter period length without compromising the required magnetic field strength. Although no practical SC-MPWs using Nb_3_Sn coils have been realized yet, the successful implementation of a Nb_3_Sn superconducting undulator (SCU) at the Advanced Photon Source (APS) in 2023 (Kesgin *et al.*, 2021 ▸; ANL, 2023 ▸) indicates its feasibility for our application. Therefore, we have selected Nb_3_Sn as the superconducting material for the V-SC-MPW. In superconducting-based IDs, horizontal winding and vertical winding are generally employed for the winding configurations. In the horizontal winding, the coils are wound in a plane parallel to the beam axis, whereas in the vertical winding, the coils are wound in a plane perpendicular to the beam axis (Gluskin & Mezentsev, 2019 ▸). While the horizontal winding is common in horizontally polarized SC-MPWs using NbTi wires, it is not suitable for V-SC-MPWs using brittle Nb_3_Sn wires because a small coil curvature radius is required for a short period design. Therefore, we adopted the vertical winding, which is able to maintain a large coil curvature radius regardless of the period length. We adopted a circular design for the coil shape because it has been used in MRI and NMR applications and has demonstrated operational stability at high currents. Fig. 1 ▸ shows a 3D model of the vertical circular winding for the V-SC-MPW.

**Figure 2:**
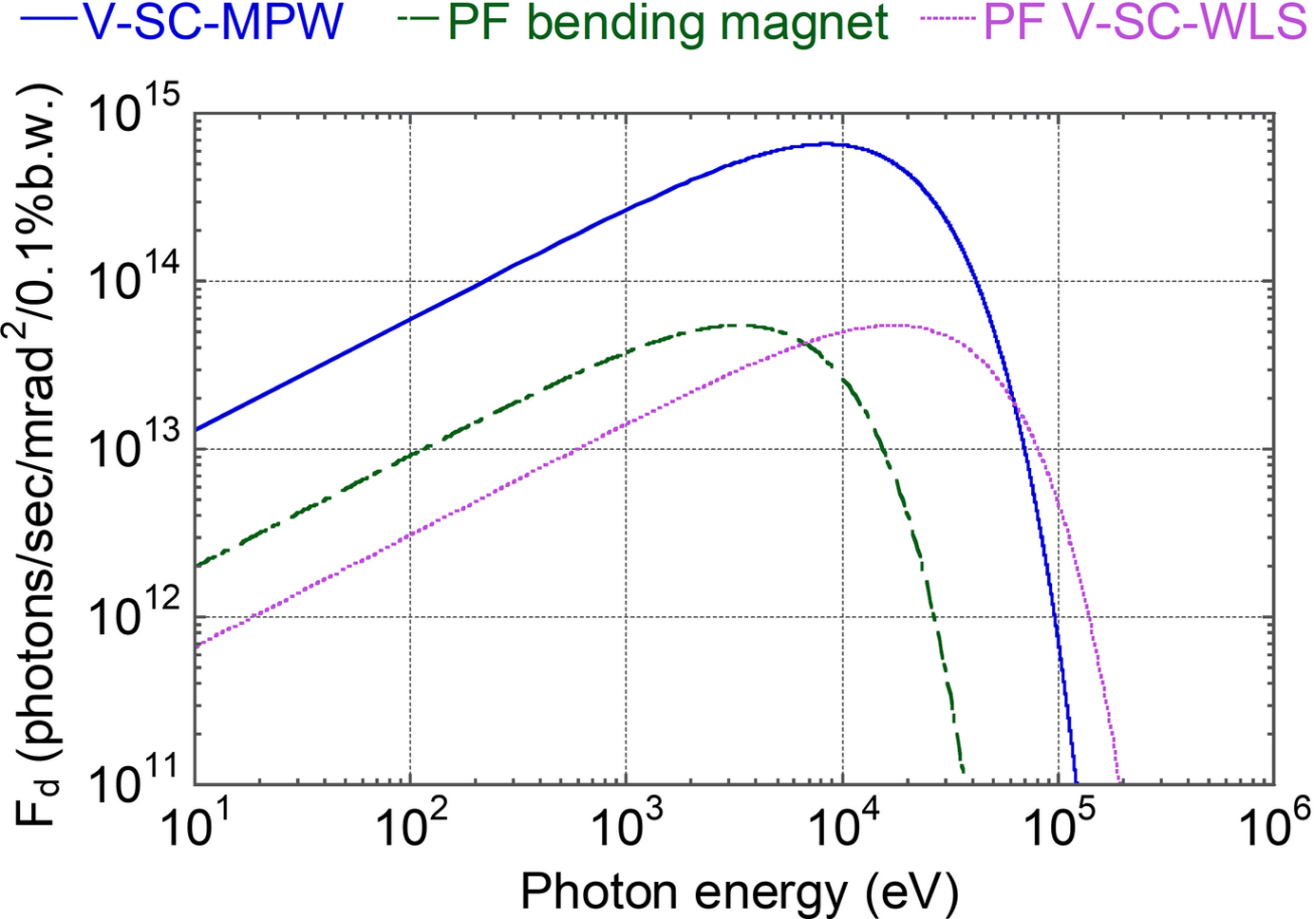
Flux density of a V-SC-MPW with a peak magnetic field of 2.44 T, a period length of 85 mm and the number of periods of 7 (V-SC-MPW-1 discussed later), which satisfies the specifications listed in Table 1 ▸. A beam energy of 2.5 GeV, a beam current of 450 mA and a horizontal beam emittance of 34.6 nm mrad are assumed, which are based on the parameters of PF. For comparison, spectra of the existing V-SC-WLS and bending magnets in PF are shown. The V-SC-MPW provides sufficiently high flux in the hard X-ray region even with the compact design.

Fig. 2 ▸ presents the radiation spectrum for a design based on the specifications listed in Table 1 ▸. For comparison, the spectra of the existing V-SC-WLS and bending magnets in PF are also shown. In this design, a flux density greater than that of the V-SC-WLS can be obtained up to approximately 60 keV. Despite the relatively small magnetic field and limited number of periods of the V-SC-MPW, it can provide a hard X-ray photon flux sufficient for practical applications.

## 3. Magnetic field calculations

To determine the extent to which the period length can be reduced while maintaining the peak magnetic field of 2–3 T in the vertical circular winding, we investigated the period length dependence of the magnetic field using the 3D magnetic field calculation code *RADIA* (Chubar *et al.*, 1998 ▸). Additionally, to confirm that the magnetic field of the V-SC-MPW with a large gap and circular coils remains sufficiently uniform, we also calculated magnetic field distributions in the horizontal and vertical directions.

### 3.1. Period length dependence of peak magnetic field

Generally, increasing the number of coil turns is effective in raising the magnetic field strength. However, as the number of turns increases in the same radial direction, the contribution to the effective magnetic field on the beam axis decreases. Thus, in a V-SC-MPW design featuring a short period and limited winding space, increasing the current density is essential for generating a high peak magnetic field. On the other hand, the maximum magnetic field inside the superconducting coil must be significantly smaller than the critical magnetic field. This requirement imposes an upper limit on the achievable period length and peak magnetic field. Increasing the current density leads to a decrease in the critical magnetic field, which subsequently affects the maximum allowable period length and peak magnetic field. Therefore, we must consider the achievable minimum period length within the range of current densities that can provide the required magnetic field. To address this, we calculated the period length dependence of the peak magnetic field and the maximum magnetic field inside the coil at different current densities. From these calculations, we examined the minimum period length and the corresponding current density necessary to achieve it.

**Figure 3:**
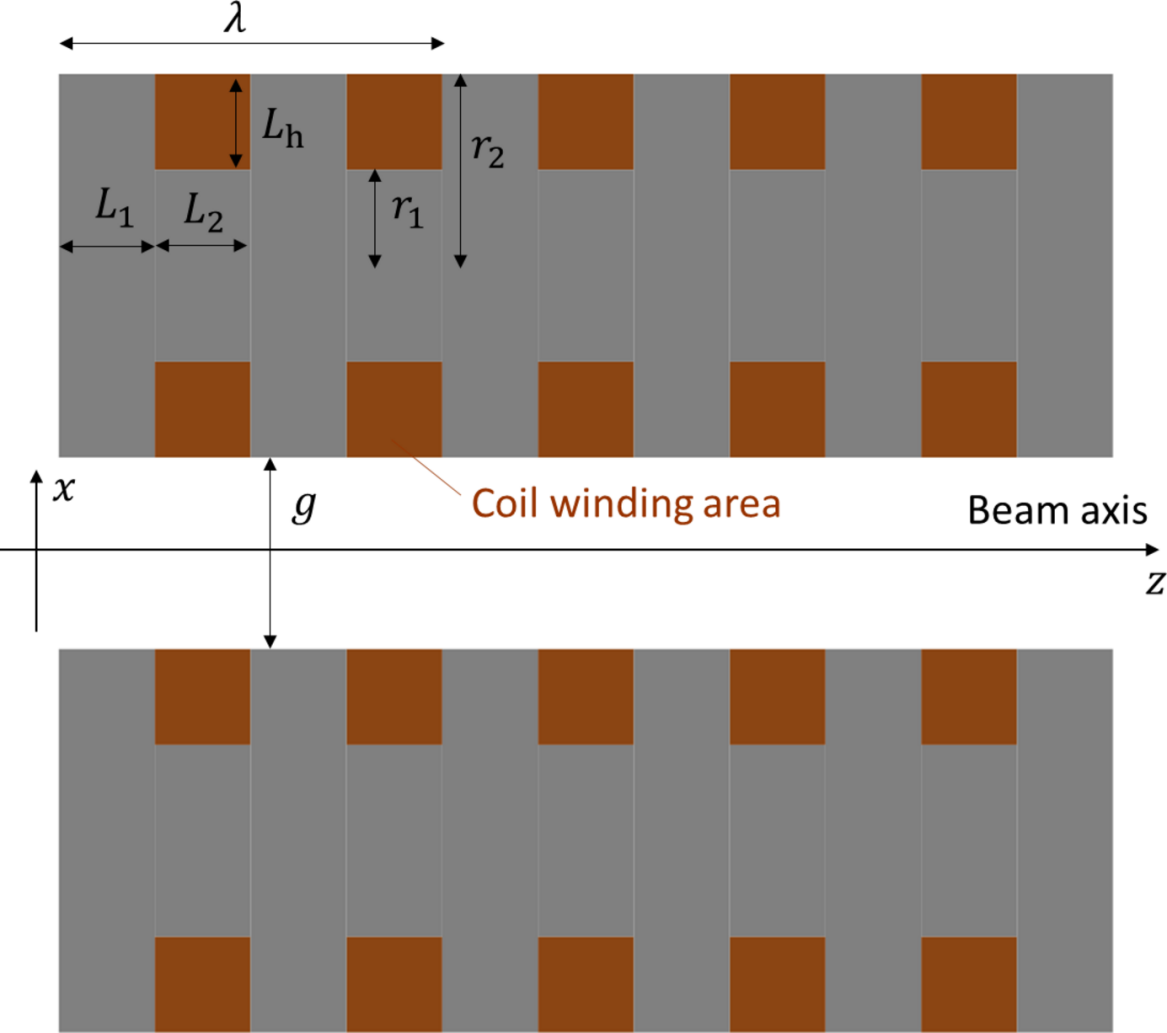
Cross-sectional view of a V-SC-MPW. The coil dimensions set as free parameters in the magnetic field calculations are also shown.

The schematic view shown in Fig. 1 ▸ is a 3D model of the V-SC-MPW in the *RADIA* calculations. Two cylindrical magnet arrays are arranged on a horizontal plane. Electron beams pass through the center of the gap between them. In the calculation, we used a coordinate system where the beam axis is *z*, the gap direction is *x* and the electron deflection direction is *y*. The free parameters for the coil size were set as the inner radius of the coil (bobbin radius) *r*_1_, the outer radius of the coil *r*_2_, the height of the coil *L*_h_, the length of the pole *L*_1_ and the length of the coil *L*_2_ (Fig. 3 ▸). Other specifications of the superconducting magnet are listed in Table 3 ▸. To reduce computational demands, the number of coils in the *z* direction was limited to 5. The non-Cu current density *J*_NC_ in the superconducting wire was set to 2000 A mm^−2^ or 1400 A mm^−2^. The copper ratio in the wire was set to 0.5, similar to the Nb_3_Sn SCU at APS (Kesgin *et al.*, 2021 ▸). The effective cross-sectional area ratio (packing factor) of the superconducting wire within the coil cross-section was set to 0.5, which is slightly low to ensure realistic estimates. In this case, the effective cross-sectional area ratio of Nb_3_Sn within the coil cross-section is 0.25. This corresponds to an engineering current density *J*_e_ = 0.25*J*_NC_. This *J*_e_ was used for the average current density over the entire coil cross-section in *RADIA* calculations. The relative permeability of the core and pole was set to 1 because the magnetic field within the coil bobbin region far exceeds iron’s saturation flux density, limiting the enhancement effect of magnetic materials. For simplicity, we assumed equal currents in all coils for the calculation. In practice, however, the currents at the ends are generally reduced to adjust the beam orbit. Since the magnetic field periodicity is not maintained at the ends, the peak magnetic field *B*_peak_ was defined as the peak value of the horizontal magnetic field at the central pole. We also calculated the magnetic field in the coil cross-section shown in Fig. 3 ▸. The maximum magnetic field inside the coils *B*_max_ was defined as the maximum field within the coil winding areas excluding the ends.

Prior to investigating the period length dependence of the magnetic fields, we examined the dependence of *B*_peak_ on the coil size and size ratio in the transverse direction (direction perpendicular to the beam axis) to determine the optimal conditions for maximizing *B*_peak_. Specifically, we first set the gap to *g* = 40 mm and the period length to λ = 80 mm. Under these conditions, we investigated the dependence of *B*_peak_ on the *r*_1_:*L*_h_ ratio and determined *r*_1_ = *L*_h_ as the optimal value. Using this condition, we next examined the dependence of *B*_peak_ on the coil radius and adopted *r*_1_ = λ/2 as the optimization condition. Finally, with these optimized parameters, we investigated the dependence on the *L*_1_:*L*_2_ ratio and selected *L*_2_/(*L*_1_ + *L*_2_) = 0.8 as the optimal setting.

**Figure 4:**
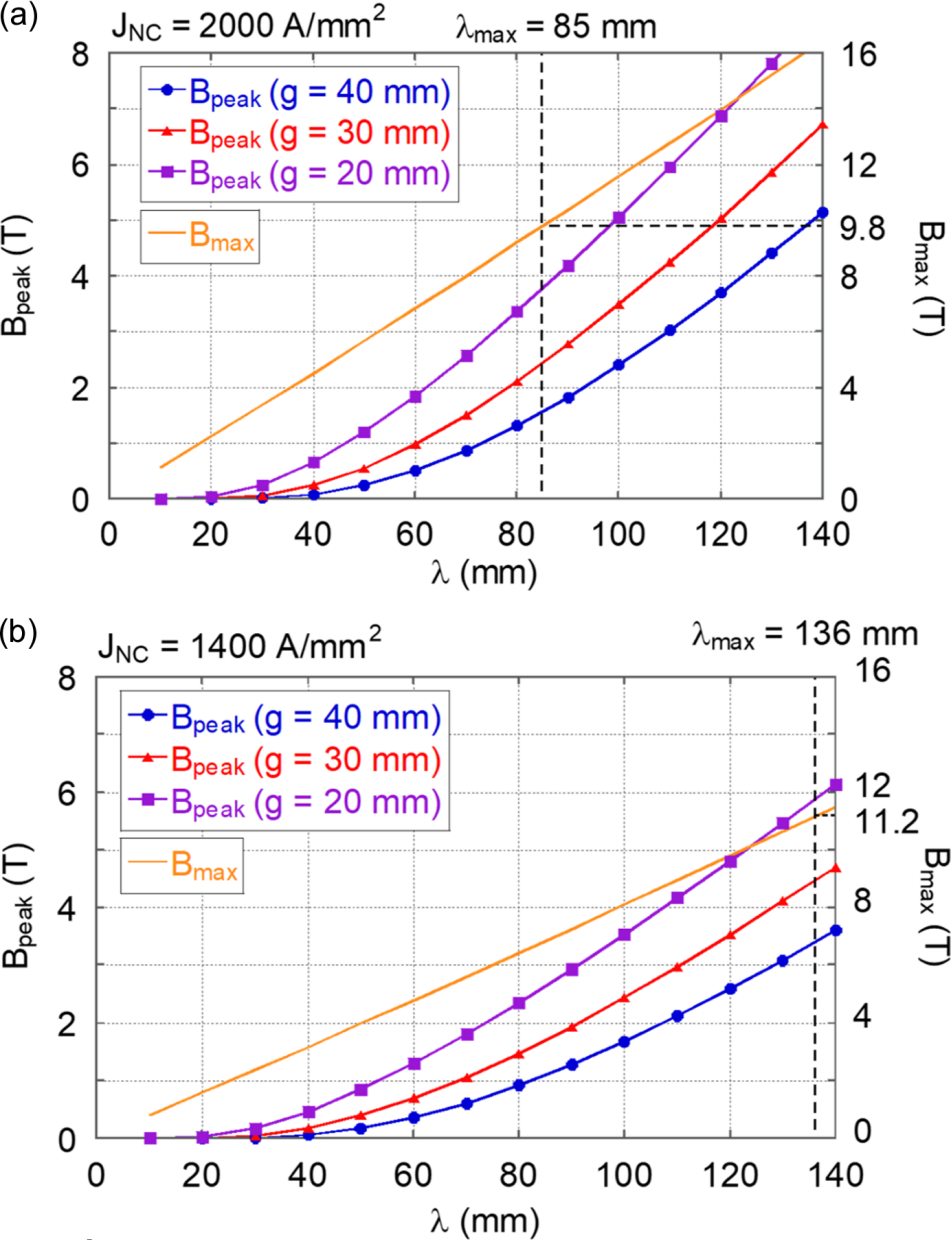
Period length dependence of the peak magnetic field *B*_peak_ and maximum magnetic field inside the coil *B*_max_. (*a*) *J*_NC_ = 2000 A mm^−2^. (*b*) *J*_NC_ = 1400 A mm^−2^. The horizontal dashed line indicates *B*_max_ corresponding to a load factor of 70%. The vertical dashed line shows the corresponding maximum allowable period length λ_max_. As the current density increases, λ_max_ and the corresponding *B*_peak_ significantly decrease.

Fig. 4 ▸ shows the calculation results of the period length dependence of *B*_peak_ and *B*_max_ under these transverse coil size conditions. The left vertical axis shows the values of *B*_peak_ and the right vertical axis shows the values of *B*_max_. Calculation results for *B*_peak_ are presented for *g* = 30 mm, along with additional cases of *g* = 20 mm and *g* = 40 mm. Since the maximum design current for Nb_3_Sn SCU is set to 80% of the critical current at APS (Kesgin *et al.*, 2021 ▸), we assumed an operation at a load factor of 70% and evaluated the corresponding maximum allowable period length λ_max_ and *B*_peak_. For the dependence of the critical magnetic field on current density *B*_c_(*J*_NC_), we used data from Bruker OST (Parrell *et al.*, 2009 ▸), which has also been employed for the SCU at APS. Based on these considerations, we evaluated λ_max_ and the corresponding *B*_peak_ for each condition, as presented in Table 4 ▸. The corresponding orbit amplitudes at a beam energy of 2.5 GeV were also calculated using the following equation and listed in the same table in parentheses. 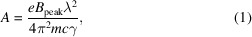
where *e* is the elementary charge, *m* is the electron mass, *c* is the speed of light and γ is the Lorentz factor.

These results clearly show that an increase in the current density leads to a significant decrease in the maximum allowable period length and peak magnetic field. Therefore, to minimize the period length, it is important to carefully select the current density such that the peak magnetic field remains within the required range. The case of *J*_NC_ = 2000 A mm^−2^, as shown in Fig. 4 ▸, corresponds to such a current density. In this case, a period length of 85 mm, a peak magnetic field of 2.44 T and an orbit amplitude of 54 µm were obtained. Therefore, V-SC-MPW can be designed with a period length of less than 100 mm and an orbit amplitude of less than 100 µm while maintaining the peak magnetic field of 2–3 T necessary for hard X-ray generation, as summarized in Table 1 ▸ in Section 2.

### 3.2. Magnetic field uniformity

**Figure 5:**
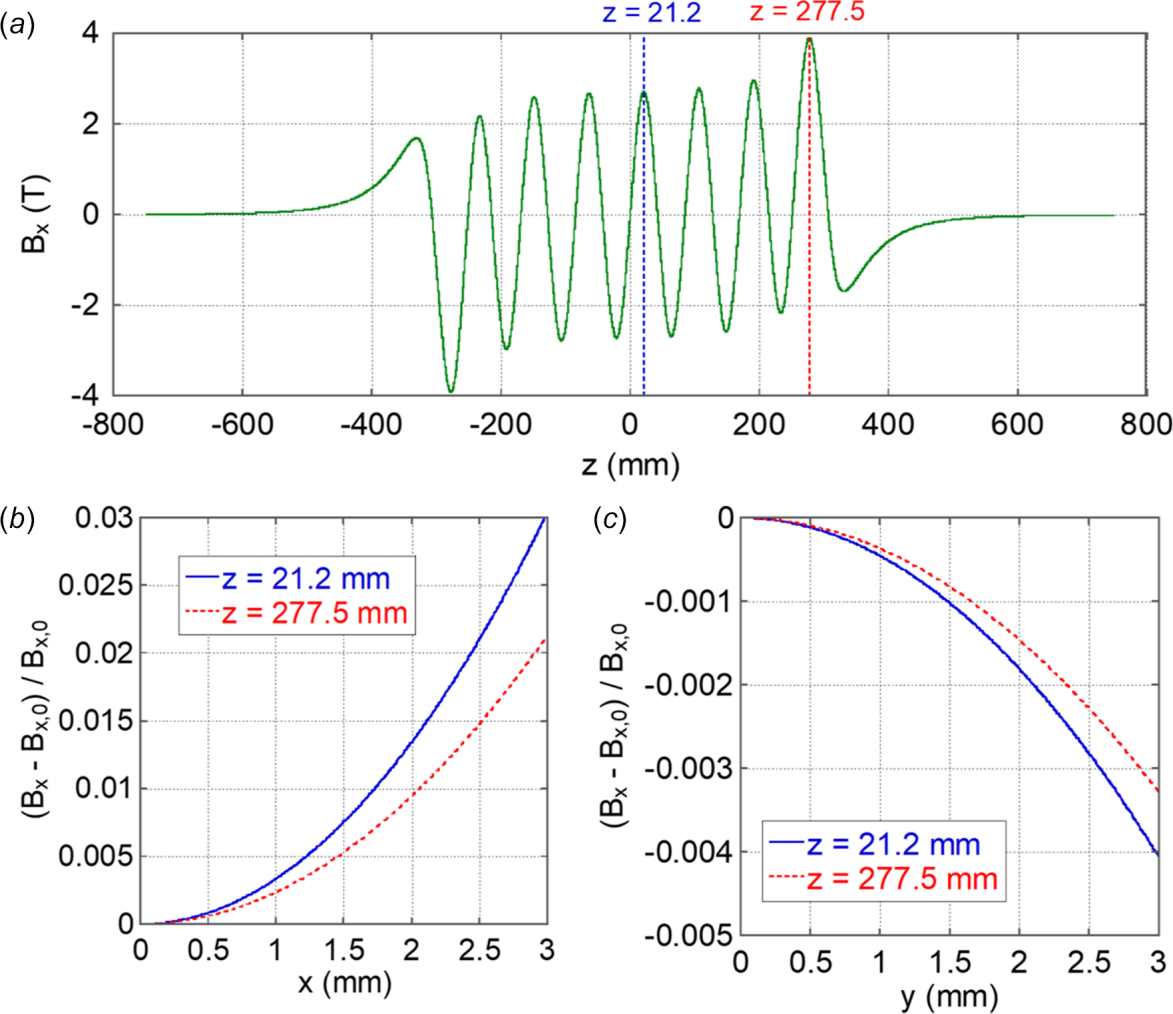
Magnetic field distribution and transverse magnetic field uniformity of the V-SC-MPW-1 with the number of coils of 15. (*a*) Magnetic field distribution along the beam axis. (*b*) Magnetic field uniformity in the horizontal direction. (*c*) Magnetic field uniformity in the vertical direction. The transverse magnetic field distributions are calculated at two peak positions: near the center (*z* = 21.2 mm) and near the end (*z* = 277.5 mm) shown in (*a*). They are shown as the relative deviation from the on-axis magnetic field *B*_*x*, 0_, given by (*B*_*x*_ − *B*_*x*,0_)/*B*_*x*,0_. Due to symmetry, only the positive side is displayed.

To investigate the magnetic field uniformity of the V-SC-MPW near the beam axis, the distributions of the horizontal magnetic field were calculated along each axis direction. Fig. 5 ▸ shows the calculation results in the beam traveling direction Fig. 5 ▸(*a*), in the horizontal direction Fig. 5 ▸(*b*) and in the vertical direction Fig. 5 ▸(*c*). The transverse magnetic field distributions are shown as the relative deviation from the on-axis magnetic field, *i.e.* (*B*_*x*_ − *B*_*x*,0_)/*B*_*x*,0_. The axis positions of the transverse distributions are *z* = 21.2 mm and *z* = 277.5 mm, which are displayed in Fig. 5 ▸(*a*). The distributions in Fig. 5 ▸ show that the magnetic field increases as the transverse position moves towards the coil in the *x* direction, while it decreases as the position moves away from the coil in the *y* direction. Since electrons have a finite beam emittance and oscillate in the vertical direction due to the wiggler field, they experience slightly shifted magnetic fields individually.

In the horizontal direction, the magnetic field deviation is approximately 0.3% at a displacement of 1 mm [see Fig. 5 ▸(*b*)]. In the case of PF-HLS, the typical r.m.s. beam size is several tens of micrometres, and most of the stored beam passes within the approximate range 200–300 µm from the beam axis. The magnetic field deviation within this range is less than approximately 0.03%. In the vertical direction, the magnetic field deviation is approximately 0.05% at 1 mm [see Fig. 5 ▸(*c*)]. The typical r.m.s. beam size in the vertical direction is several micrometres. In addition, the beam oscillates with an amplitude of several tens of micrometres from the beam axis. Therefore, most of the beam passes within 100–200 µm. The magnetic field deviation in this range is less than approximately 2 × 10^−3^%.

Because the magnetic field variations within these beam size ranges are minimal, and the V-SC-MPW functions as a broadband light source, these deviations have a negligible impact on the radiation spectrum. While a detailed evaluation of the impact on the global beam orbit is beyond the scope of this study, the local magnetic field deviations are not significantly larger than those in typical IDs. Another important consideration is the integrated defocusing effect arising from field roll-off. Particle tracking simulations indicate a vertical integrated quadrupole strength (Δ*kL*) of approximately 0.016 m^−1^ (equivalent to *B*ρΔ*kL* = 0.13 T at 2.5 GeV) due to the *B*_*x*_ field roll-off. This corresponds to a vertical tune shift of approximately 0.0067 in the PF-HLS normal cell (described in Section 4), which is considered manageable with standard beam correction techniques. Therefore, although features like the large gap and circular coils in the V-SC-MPW are not optimal for magnetic field uniformity, the overall impact of the resulting non-uniformities is expected to be correctable and remains acceptable for practical applications.

## 4. Impact on beam emittance

To investigate the impact on beam emittance for designs with orbit amplitudes around 100 µm, we considered the following parameter sets based on Table 4 ▸ and calculated the resulting emittance growth in PF-HLS. V-SC-MPW-1: λ = 85 mm, *B*_peak_ = 2.44 T (*A* = 54 µm), *N* = 7, *P* = 7.0 kW; V-SC-MPW-2: λ = 136 mm, *B*_peak_ = 4.49 T (*A* = 252 µm), *N* = 7, *P* = 38.0 kW; where *N* is the number of periods and *P* is the average radiation power.

**Figure 6:**
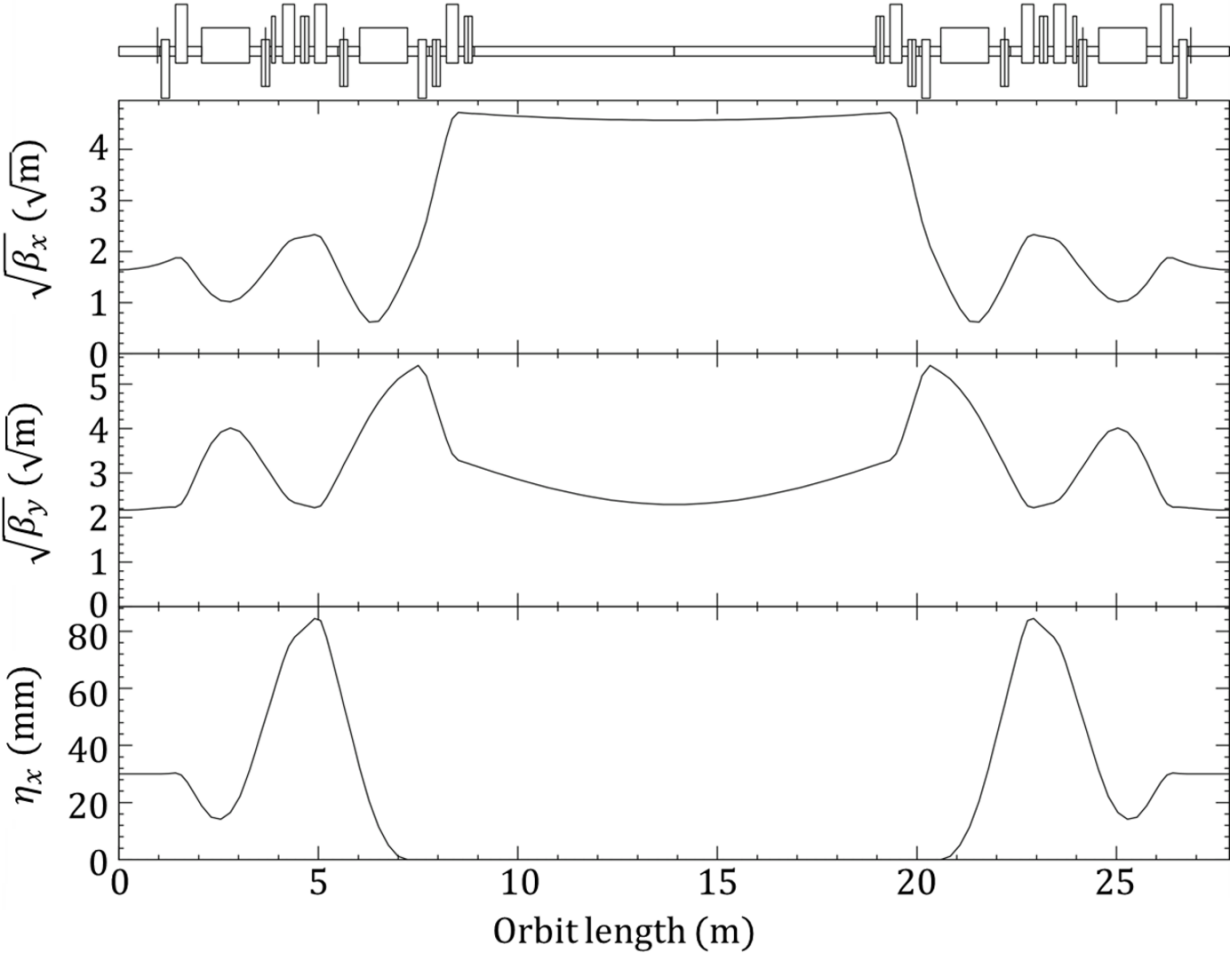
Optics of a normal cell with a 10 m straight section in PF-HLS. The figure shows three optical functions: 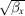 and 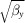 (square root of the horizontal and vertical betatron functions, respectively) and η_*x*_ (horizontal dispersion function). The emittance change calculations assumed the installation of a V-SC-MPW in either the 10 m achromatic straight section or the 2 m non-achromatic straight section located at both ends of the cell.

Since the effects of quantum excitation by photon emissions depend not only on the parameters of the ID but also on the beam optics, the emittance change varies depending on the location of the ID within the ring. PF-HLS consists of four basic cell types. Each cell includes a 5 m or 10 m achromatic straight section and a 2 m non-achromatic straight section, where IDs can be installed (High Energy Accelerator Research Organization, 2024 ▸). Fig. 6 ▸ shows the optics of one of the four basic cell types, specifically a normal cell that includes a 10 m straight section. To evaluate the influence of horizontal dispersion, we analyzed the emittance change assuming that a single V-SC-MPW was introduced into either the 10 m straight section (with β_*x*_ = 20.9 m, β_*y*_ = 5.3 m and η_*x*_ = 0 mm at its center) or the 2 m straight section (with β_*x*_ = 2.7 m, β_*y*_ = 4.7 m and η_*x*_ = 30 mm at its center) of this cell.

Table 5 ▸ shows the calculated equilibrium emittances ɛ_*x*_ and ɛ_*y*_, as well as the relative energy spreads σ_*E*_/*E* for the cases without an ID, with V-SC-MPW-1 and with V-SC-MPW-2. These results were obtained by analytically calculating the synchrotron radiation integrals (Helm *et al.*, 1973 ▸; Katoh & Kamiya, 1987 ▸), assuming a sinusoidal magnetic field distribution for the IDs. For simplicity, the calculations were performed with zero beam coupling.

The emittance change depends on the Twiss parameters and the dispersion function. Since the beam deflection direction in the V-SC-MPW is vertical, the horizontal emittance change is significantly affected by the dispersion function of the straight section. The horizontal emittance decreases in the case of the 10 m straight section because the effect of radiation damping dominates. In contrast, it increases in the case of the 2 m straight section due to stronger quantum excitation in the dispersive section. In the vertical direction, the emittance change does not strongly depend on the installation location because the effects of quantum excitation are largely influenced by the dispersion created by the ID itself.

In the case of V-SC-MPW-1 with the small orbit amplitude design of 54 µm, the vertical emittance growth is as small as approximately 1 pm rad. Even in the 2 m straight section, the horizontal emittance growth is limited to 15.6 pm rad. This result suggests that V-SC-MPW-1 can be installed in any straight section. In contrast, for V-SC-MPW-2 with the orbit amplitude of 252 µm, the vertical emittance increases significantly to ɛ_*y*_ = 97.6 pm rad even in the 10 m straight section. This substantial increase makes its introduction to PF-HLS impractical. A comparison of the calculation results of the two designs indicates that the emittance growth is highly sensitive to changes in the period length and orbit amplitude. This highlights the importance of a short-period design, such as V-SC-MPW-1, with an orbit amplitude on the order of tens of micrometres.

The impact on emittance depends on various factors, including beam parameters and ring optics. Therefore, it is difficult to uniformly determine an upper limit for the orbit amplitude that ensures emittance growth remains below a certain value. However, the calculation results in Table 5 ▸ suggest that an orbit amplitude of 100 µm serves as an effective guideline for rings with an emittance comparable to or greater than PF-HLS. Furthermore, the parameters in Table 1 ▸ provide reasonable design criteria for the overall V-SC-MPW. Therefore, the V-SC-MPW that meets these requirements is a versatile ID that enables the use of vertically polarized hard X-rays in modern low-emittance rings operating at intermediate beam energies.

## 5. Summary

We have investigated a vertically polarized superconducting multipole wiggler with a short-period design, focusing on reducing beam emittance growth. Using Nb_3_Sn wires, which have approximately 10 times the critical current density of conventional NbTi, and adopting a vertical circular winding, we have shown that it is possible to achieve a peak magnetic field of 2–3 T necessary for hard X-ray generation while keeping the period length below 100 mm and the orbit amplitude below 100 µm. We also confirmed that sufficient transverse magnetic field uniformity is ensured. Although the impact of quantum excitation on beam emittance depends on beam parameters and ring optics, we considered a specific case where a V-SC-MPW with a peak magnetic field of 2.44 T, a period length of 85 mm (orbit amplitude of 54 µm under a beam energy of 2.5 GeV), and seven periods is introduced into a straight section with a dispersion function of 30 mm and betatron functions of β_*x*_ = 2.7 m and β_*y*_ = 4.7 m in PF-HLS. The estimated emittance growths in this case were 15.6 pm rad horizontally and 1.0 pm rad vertically. These results indicate that the proposed V-SC-MPW is a compact, vertically polarized hard X-ray source with minimal impact on the stored beam. It shows great potential as a versatile ID to facilitate the use of vertically polarized hard X-rays in modern low-emittance rings with intermediate beam energies.

## Figures and Tables

**Table 1:** Required features and target parameters of the discussed V-SC-MPW

Polarization	Vertical linear
Gap (horizontal) *g*	30 mm
Peak magnetic field *B*_peak_	2–3 T
Period length λ	<100 mm
Orbit amplitude of electrons *A*	<100 µm
Number of periods *N*	<10
Total magnet length	<1 m
Radiation power *P*	<10 kW
Superconducting material	Nb_3_Sn
Winding scheme	Vertical winding
Coil shape	Circle

**Table 2:** Main beam parameters of PF-HLS (High Energy Accelerator Research Organization, 2024 ▸)

Beam energy	2.5 GeV
Circumference	749.5 m
RF voltage	1.6 MV
SR energy loss per turn	0.222 MeV
Momentum compaction factor	3.24 × 10^−5^
Horizontal/vertical tune	47.865/16.655
Stored beam current	500 mA
Natural horizontal emittance	208 pm rad
Relative energy spread	7.4 × 10^−4^
Natural bunch length	4.72 ps
Beam coupling	1% (assumed)

**Table 3:** Specifications of the superconducting wire and coil for the investigation of the period length dependence

Number of coils	5
Non-Cu current density *J*_NC_	2000 or 1400 A mm^−2^
Cu ratio	0.5
Packing factor	0.5
Engineering current density *J*_e_	500 or 350 A mm^−2^
Relative permeability	1

**Table 4:** Maximum allowable period length (λ_max_) and the corresponding peak magnetic field (*B*_peak_) The values of orbit amplitude (*A*) at each period length and magnetic field are shown in parentheses.

*J*_NC_	2000 A mm^−2^	1400 A mm^−2^
*B*_c_(*J*_NC_)	14 T	16 T
0.7*B*_c_(*J*_NC_)	9.8 T	11.2 T
λ_max_	85 mm	136 mm
*B*_peak_(*A*)		
*g* = 40 mm	1.57 T (34 µm)	3.42 T (192 µm)
*g* = 30 mm	2.44 T (54 µm)	4.49 T (252 µm)
*g* = 20 mm	3.78 T (83 µm)	5.90 T (331 µm)

**Table 5:** Beam emittances (ɛ_*x*_, ɛ_*y*_) and relative energy spread (σ_*E*_/*E*) in PF-HLS in the case where a single V-SC-MPW is installed The calculation results are shown for two installation locations: a 10 m achromatic straight section and a 2 m non-achromatic straight section. The values in parentheses indicate the differences from the case without the ID. In V-SC-MPW-2 with the orbit amplitude of 252 µm, the emittance degrades significantly, but in V-SC-MPW-1, with the small orbit amplitude design of 54 µm, the emittance growth is kept small

	No ID	V-SC-MPW-1		V-SC-MPW-2	
Location		10 m section	2 m section	10 m section	2 m section
ɛ_*x*_ (pm rad)	208.1	202.2 (−5.9)	223.7 (+15.6)	179.7 (−28.4)	370.3 (+162.2)
ɛ_*y*_ (pm rad)	0	1.3	1.0	97.6	73.4
σ_*E*_/*E* (×10^−3^)	0.742	0.792 (+0.051)		1.117 (+0.375)	
